# Supplementary material for: Adaptation by copy number variation increases insecticide resistance in the fall armyworm
Source: Commun Biol. 2020 Nov 12;3:664. doi: 10.1038/s42003-020-01382-6 (PMC7661717; doi:10.1038/s42003-020-01382-6)
Supplement: Supplementary file 9 — Reporting Summary [file 42003_2020_1382_MOESM9_ESM.pdf]

nature research

Corresponding author(s): Kiwoong NAM

Last updated by author(s): Oct 5, 2020

Reporting Summary

Nature Research wishes to improve the reproducibility of the work that we publish. This form provides structure for consistency and transparency in reporting. For further information on Nature Research policies, see our Editorial Policies and the Editorial Policy Checklist.

Statistics

For all statistical analyses, confirm that the following items are present in the figure legend, table legend, main text, or Methods section.

n/a	Confirmed	
☐	☒	The exact sample size (*n*) for each experimental group/condition, given as a discrete number and unit of measurement
☐	☒	A statement on whether measurements were taken from distinct samples or whether the same sample was measured repeatedly
☐	☒	The statistical test(s) used AND whether they are one- or two-sided<br>*Only common tests should be described solely by name; describe more complex techniques in the Methods section.*
☐	☒	A description of all covariates tested
☐	☒	A description of any assumptions or corrections, such as tests of normality and adjustment for multiple comparisons
☐	☒	A full description of the statistical parameters including central tendency (e.g. means) or other basic estimates (e.g. regression coefficient) AND variation (e.g. standard deviation) or associated estimates of uncertainty (e.g. confidence intervals)
☐	☒	For null hypothesis testing, the test statistic (e.g. *F*, *t*, *r*) with confidence intervals, effect sizes, degrees of freedom and *P* value noted<br>*Give P values as exact values whenever suitable.*
☒	☐	For Bayesian analysis, information on the choice of priors and Markov chain Monte Carlo settings
☒	☐	For hierarchical and complex designs, identification of the appropriate level for tests and full reporting of outcomes
☒	☐	Estimates of effect sizes (e.g. Cohen's *d*, Pearson's *r*), indicating how they were calculated

Our web collection on statistics for biologists contains articles on many of the points above.

Software and code

Policy information about availability of computer code

Data collection	We used GATK-4.0.11.0 and CNVcaller to identify genetic variants.
Data analysis	We used vcftools 0.1.13, Picard 1.9, BinGO 3.0.3, POLO-PLUS, and admixture 1.3.0. We also used custom perl/R scripts to process result files from the software. These custom perl and R scripts are available on request.

For manuscripts utilizing custom algorithms or software that are central to the research but not yet described in published literature, software must be made available to editors and reviewers. We strongly encourage code deposition in a community repository (e.g. GitHub). See the Nature Research guidelines for submitting code & software for further information.

Data

Policy information about availability of data

All manuscripts must include a data availability statement. This statement should provide the following information, where applicable:

- Accession codes, unique identifiers, or web links for publicly available datasets
- A list of figures that have associated raw data
- A description of any restrictions on data availability

The resequencing data in this study is available at NCBI SRA (PRJNA494340, PRJNA577869).
The reference genome assembly is available at NCBI (JACWZW000000000) together with raw PacBio reads and Hi-C data (PRJNA662887). The reference genome is also available at BIPAA (www.bipaa.genouest.org/sp/spodoptera_frugiperda).
VCF files generated in this study is available at Zenodo (doi:10.5281/zenodo.4024047). We also provide all scripts used in this study from Zenodo (doi:10.5281/zenodo.4024357).
We provide all raw data used to generate figure at Supplementary Material.

Field-specific reporting

Please select the one below that is the best fit for your research. If you are not sure, read the appropriate sections before making your selection.

☐ Life sciences ☐ Behavioural & social sciences ☒ Ecological, evolutionary & environmental sciences

For a reference copy of the document with all sections, see nature.com/documents/nr-reporting-summary-flat.pdf

Ecological, evolutionary & environmental sciences study design

All studies must disclose on these points even when the disclosure is negative.

Study description	The data is factorial, based on the sampling sites or on the host-plant strains.
Research sample	sfC and sfR are host-plant strains, meaning that these two groups have a different preference in host-plant.<br>MS and PR are groups sampled from Mississippi and Puerto Rico. These two groups have different levels of insecticide resistance.<br><br>We collected samples at the larval stage. To remove contaminants, we raised the larvae to adults, and we extracted gDNA from the thorax.
Sampling strategy	We collected samples based on randomization. And we used all samples available.
Data collection	We collected samples from crop fields by hand. The collection was performed by Carlos Blanco for another study (Blanco, C. A. et al. Susceptibility of isofamilies of Spodoptera frugiperda (Lepidoptera: Noctuidae) to Cry1Ac and Cry1Fa proteins of Bacillus thuringiensis. Southwestern Entomologist 35, 409–416 (2010)), and we re-used remaining samples for this study.
Timing and spatial scale	Sampling from Puerto Rico was performed at Santa Isabel on 8th-Oct-2009. Sampling from Mississippi was performed at Stoneville on 2nd-Oct-2009. The sampling was performed for another study, which aims at studying Bt-resistance (Blanco, C. A. et al. Susceptibility of isofamilies of Spodoptera frugiperda (Lepidoptera: Noctuidae) to Cry1Ac and Cry1Fa proteins of Bacillus thuringiensis. Southwestern Entomologist 35, 409–416 (2010)). However, we can re-use it to study chemical resistance in this study as well because differential resistance to chemical resistance was demonstrated in our analysis.
Data exclusions	We did not exclude any samples.
Reproducibility	gDNA is still available from all the samples used in this study. Therefore, we can re-generate all resequencing data as well.
Randomization	We allocated samples to MS and PR according to sampling sites.<br>In addition, we allocated samples to sfC and sfR from mitochondrial markers.
Blinding	In order to test the effect of grouping, we randomly generated two groups with 1000 replications to test if the observed difference between groups can be explained by chance.

Did the study involve field work? ☒ Yes ☐ No

Field work, collection and transport

Field conditions	Sampling was performed corn fields.
Location	Santa Isabel (Puerto Rico) and Stoneville (Mississippi).
Access & import/export	The access was provided from co-authors in this paper: Blanco, C. A. et al. Susceptibility of isofamilies of Spodoptera frugiperda (Lepidoptera: Noctuidae) to Cry1Ac and Cry1Fa proteins of Bacillus thuringiensis. Southwestern Entomologist 35, 409–416 (2010).<br><br>As the sampling was performed 2009, Nagoya protocol is not applicable.
Disturbance	We are not aware of any disturbance.

Reporting for specific materials, systems and methods

We require information from authors about some types of materials, experimental systems and methods used in many studies. Here, indicate whether each material, system or method listed is relevant to your study. If you are not sure if a list item applies to your research, read the appropriate section before selecting a response.

Materials & experimental systems

n/a	Involved in the study	
☒	☐	Antibodies
☒	☐	Eukaryotic cell lines
☒	☐	Palaeontology and archaeology
☐	☒	Animals and other organisms
☒	☐	Human research participants
☒	☐	Clinical data
☒	☐	Dual use research of concern

Methods

n/a	Involved in the study	
☒	☐	ChIP-seq
☒	☐	Flow cytometry
☒	☐	MRI-based neuroimaging

Animals and other organisms

Policy information about studies involving animals; ARRIVE guidelines recommended for reporting animal research

Laboratory animals	Benzon strain, 456LSD4 strain (Spodoptera frugiperda)
Wild animals	Larvae of Spodoptera frugiperda was collected from corn fields on 8th-Oct-2009 at Santa Isabel in Puerto Rico and on 2th-Oct-2009 at Stoneville in Mississippi. The larvae was raised to adults in a lab, and we extracted gDNA from the thorax.
Field-collected samples	The samples are kept in ethanol at -80°C befoe extracting gDNA.
Ethics oversight	No ethical approval is required for this study.

Note that full information on the approval of the study protocol must also be provided in the manuscript.
